# Origin of the Chinese word for “cell”: an unusual but wonderful idea of a mathematician

**DOI:** 10.1007/s13238-020-00790-y

**Published:** 2020-09-21

**Authors:** He Zhang

**Affiliations:** 1 School of Marxism, Bengbu Medical College, Bengbu, 233030 China; 2 Department for the History of Science and Scientific Archaeology, University of Science and Technology of China, Hefei, 230026 China

In today’s biology and even the whole field of natural science, “cell” is undoubtedly a central concept. Even in everyday life, it is a well-known common word. Then, who could have thought that “xibao” (细胞), the Chinese word for “cell”, was not coined by a biologist, but was instead the inspired idea of a mathematician! This mathematician is Shanlan Li (李善兰), an exceptional scholar from the late Qing dynasty.

**Figure 1:**
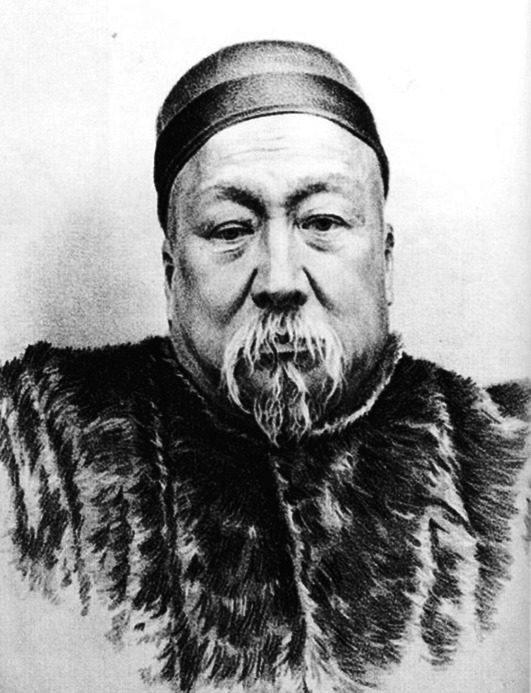
**Shanlan Li (1811–1882)**

Shanlan Li (Fig. 1) was born on January 2nd, 1811 in Haining, Zhejiang Province. He was obsessed with mathematics since he was a child. When he was 9 years old, he found the *Nine Chapters on the Mathematical Art* on the shelf. He immediately read this ancient Chinese mathematical masterpiece, and became enamored with mathematics. At the age of 14, he taught himself the first six volumes of the Chinese version of *Euclid*’*s Elements*. After the age of 30, Li started in-depth study of mathematics, and began to write books on it. By the age of 40, he had published several mathematical monographs and become a renowned mathematician. His mathematical research represented the most advanced level of Chinese mathematics at the time. Li was also a pioneer of calculus in China (Wang, 2000).

**Figure 2:**
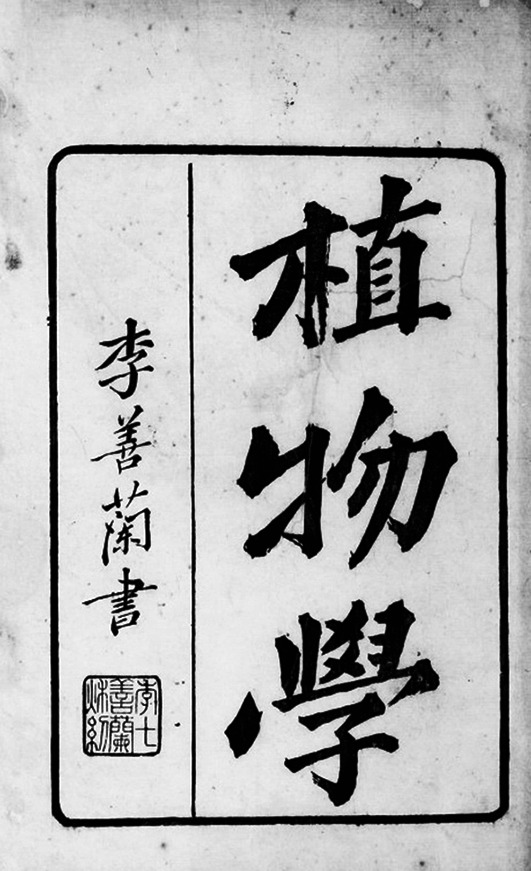
***Chih-wu hsüeh*, by Alexander Williamson, Joseph Edkins, and Shanlan Li in 1858**

In the summer of 1852, Li presented his mathematical works to foreign missionaries at the London Missionary Society Press (Mo Hai Shu Kuan) in Shanghai, and was commended by Alexander Wylie and colleagues. From there on, Li cooperated with Alexander Wylie, Joseph Edkins and others to translate western scientific writings, successively publishing a number of important translations. In 1858, the Press published a translation titled *Chih-wu hsüeh* (Botany) (Fig. 2), which was compiled and translated by Alexander Williamson and Joseph Edkins, two British missionaries, together with Shanlan Li. As the first translated work on modern Western botany, this translation catalyzed the spread of modern Western botany in China. The Chinese word for cell, “xibao” (细胞), originated in this translation.

**Figure 3:**
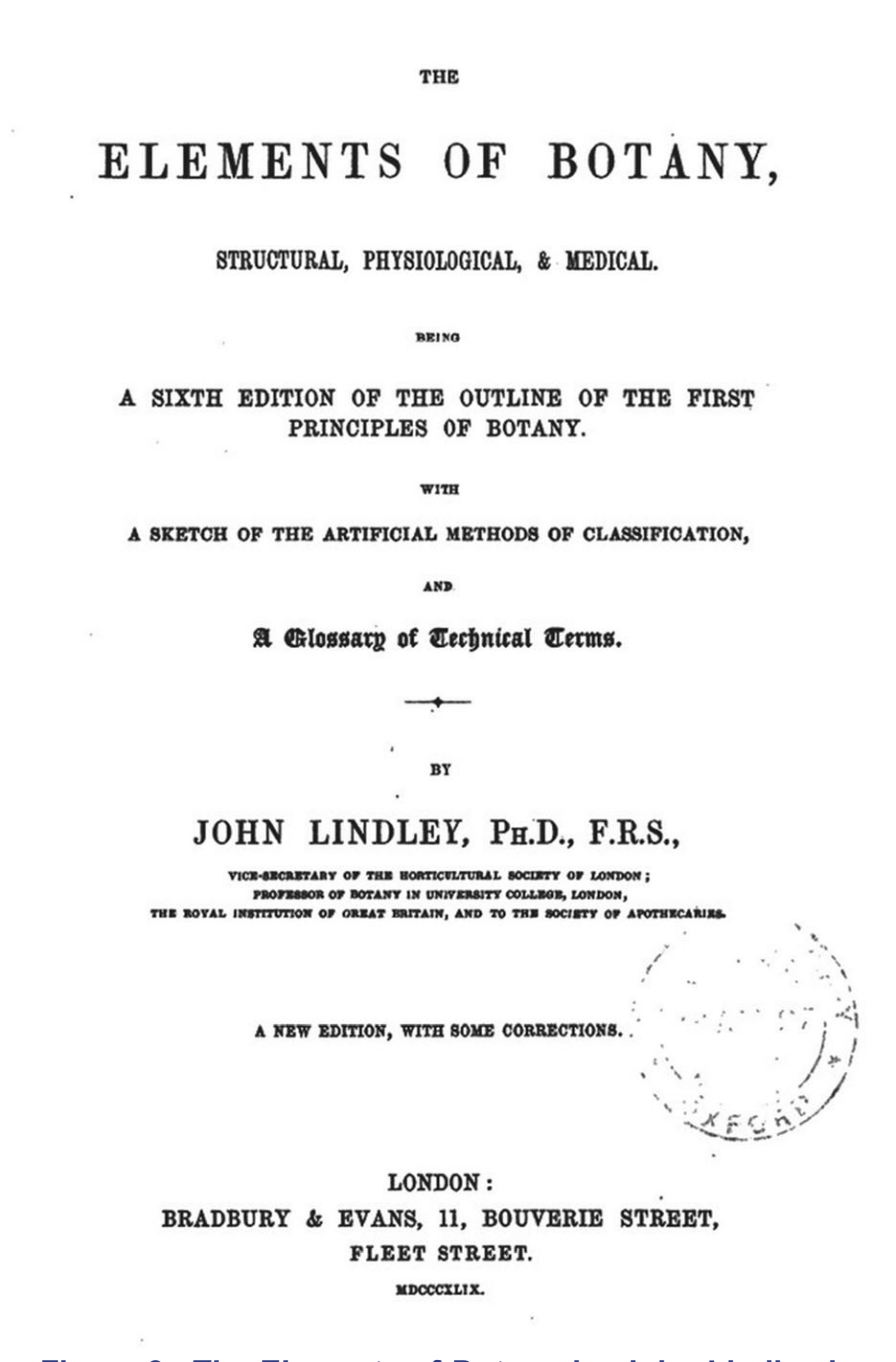
*The Elements of Botany*, by John Lindley in 1849

For a long time, the original foreign source of *Chih-wu hsüeh* (Williamson et al., 1858) had generally been regarded as a problem. Recently, studies concluded that at least four original sources were compiled into *Chih-wu hsüeh*, including Lindley’s 1847 or 1849*The Elements of Botany*, John Hutton Balfour’s 1851*Phyto-Theology*, Lindley’s 1853*The Vegetable Kingdom*, and Volume 1 of *Chambers*’ *Information for the People* published by William and Robert Chambers in 1848 (Zhang, 2018). The edition of Lindley’s *The Elements of Botany* was identified as that from 1849 (Fig. 3) by the author’s textual research.

According to the studies of linguists, most free translations of foreign words in modern Chinese were not invented by Chinese people, but borrowed from the original translations of Japanese authors who used Chinese characters (Wang, 2012). Even common everyday words of modern Chinese such as “science”, “society”, “nation” and “religion” come from the translations of Western languages by Japanese scholars, let alone a large number of academic Chinese words like “atom” and “molecule”. However, quite a few words were originally translated by Chinese authors, with “cell” being a notable representative.

**Figure 4:**
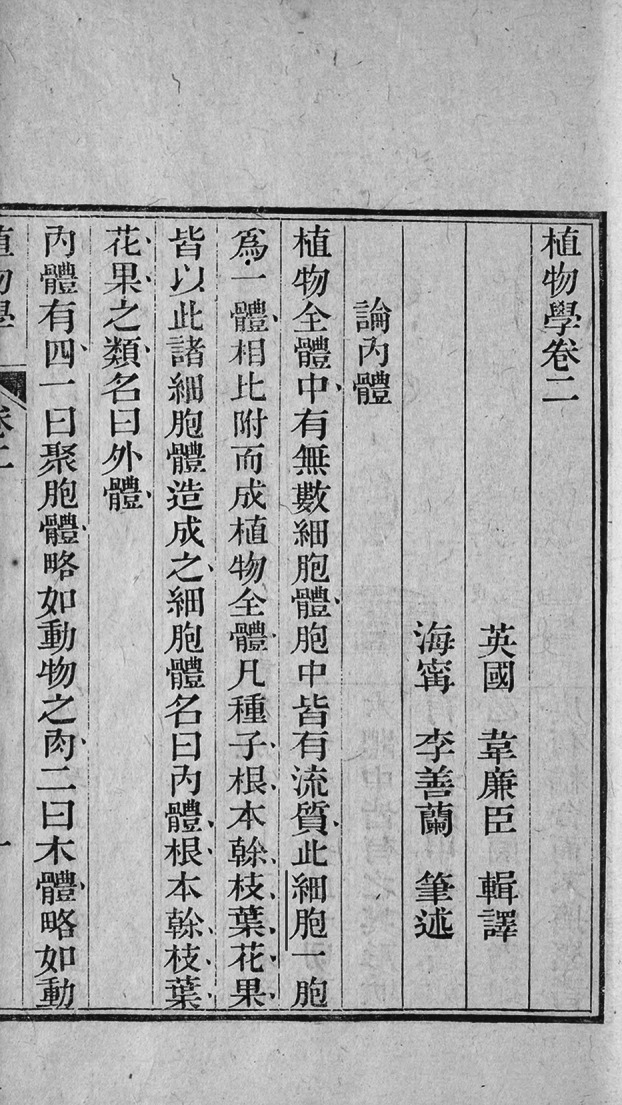
Page 1a of volume 2 of *Chih-wu hsüeh*

**Figure 5:**
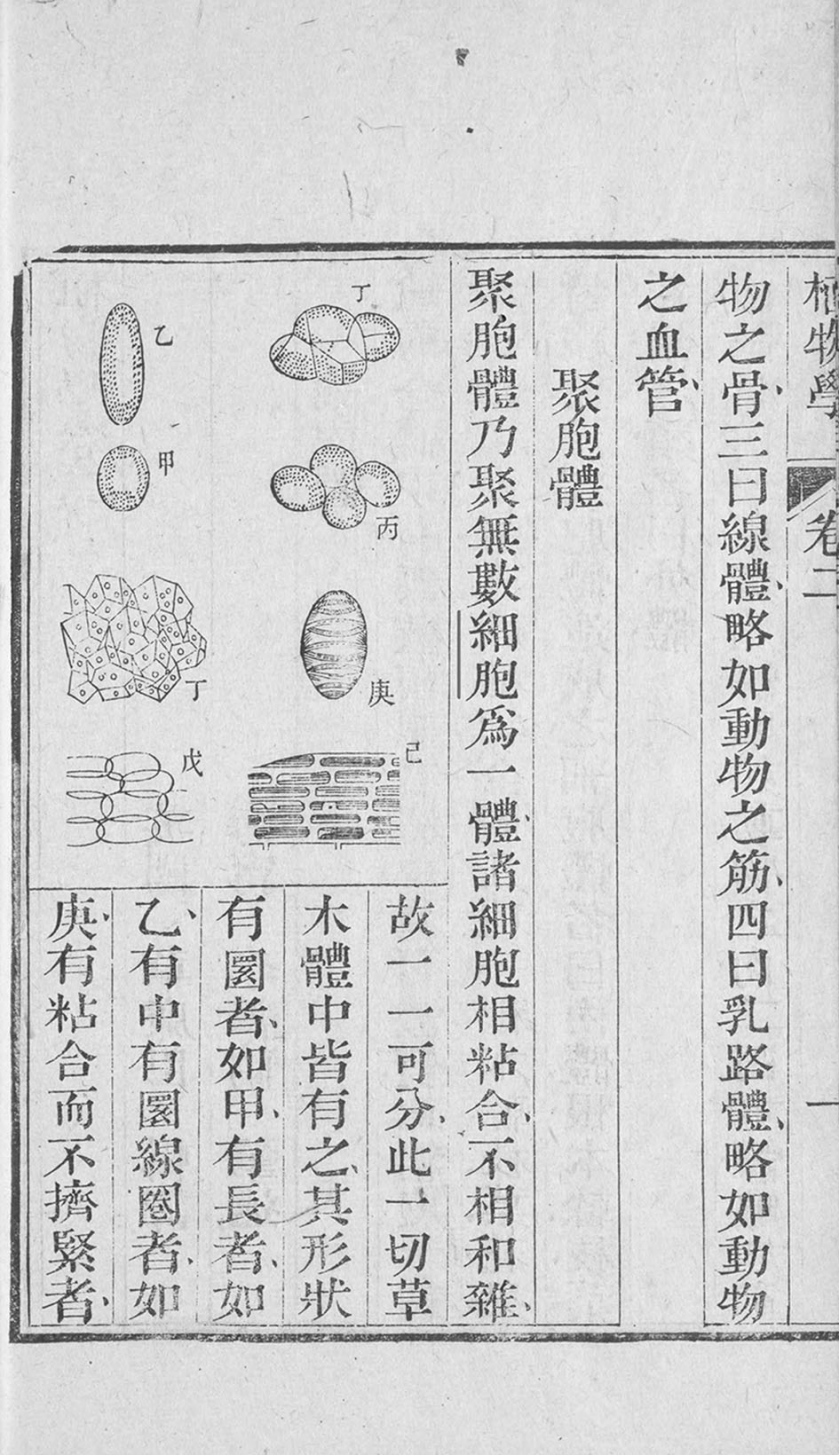
Page 1b of volume 2 of *Chih-wu hsüeh*

**Figure 6:**
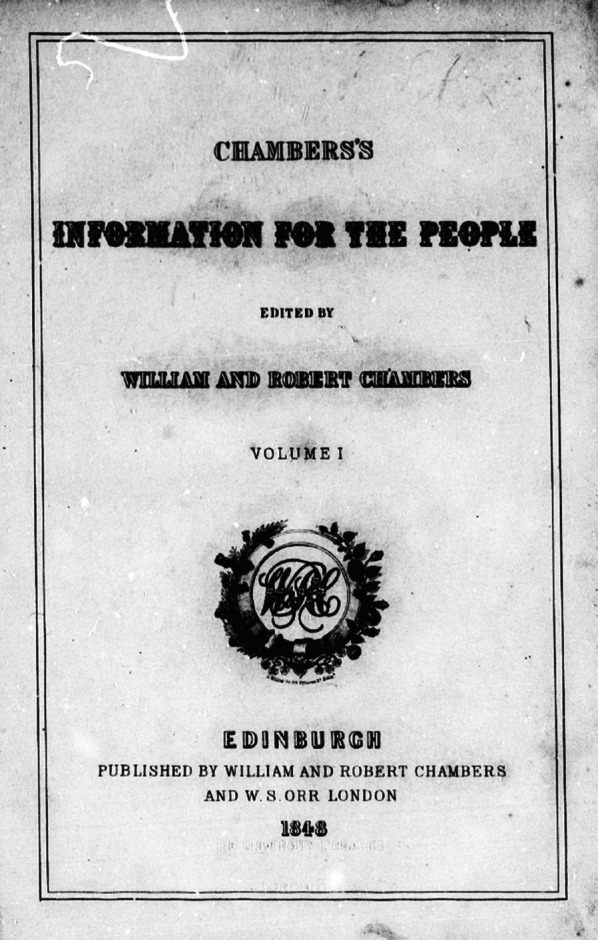
Volume 1 of *Chambers’ Information for the People*, by William and Robert Chambers in 1848

Academics mostly agree on the origin of the Chinese word “xibao” (细胞) in *Chih-wu hsüeh*, the first translated work in Chinese on modern Western botany. The description at the beginning of the second volume of the book (Fig. 4), “此细胞一胞为一体, 相比附而成植物全体” (Each cell is an independent unit, and they are interconnected to constitute a whole plant.), is often considered the paragraph in which “cell” was translated as “细胞” for the first time. At first glance, this statement is not problematic, because this is indeed where the Chinese characters of word “cell” shows up for the first time in the book *Chih-wu hsüeh*. However, by referring to the English original, we can find the source text “Each vesicle is a distinct individual, cohering with the vesicle with which it is in contact.” is located on page 3 of Lindley’s 1849 *The Elements of Botany*. The Chinese characters of word “cell” here corresponds to the English word “vesicle” instead of “cell”. In fact, the sentence “聚胞体乃聚无数细胞为一体, 诸细胞相粘合” (The aggregation integrates innumerable cells into one. These cells are bonded with each other.) on Page 1b of the second volume (Fig. 5) is the real origin translation of “cell” as Chinese characters, and its source text is not Lindley’s 1849 *The Elements of Botany*, but rather “It consists of a great number of cells of irregular shape, which adhere together.” on page 69 of Volume 1 of *Chambers*’ *Information for the People* published in 1848 (Fig. 6).

A popular viewpoint was that Li understood “cell” as “a small body with a membrane”, so he should have translated “cell” as “小胞” in Chinese. Since Li was from Haining, Zhejiang Province, and “small” (小) is used interchangeably with “thin” (细) in his dialect, he finally translated “cell” as “细胞”. This is why the Chinese word “细胞” was not recognized by the public for a long time (Yang, 2006). However, one meaning of “细” was “small or thin” in ancient Chinese (Xu, 2007), which conforms to the meaning of “cell”. Thus, it is not unsubstantiated to translate “cell” as “细胞”, and Li was unnecessarily considered to have been mainly influenced by his dialect. The main reason why the Chinese word “cell” was not recognized by the public for a long time is that the book *Chih-wu hsüeh* was not very popular in Chinese society of the late Qing dynasty and had little influence. However, the book was spread to Japan several years after it was published, and had a great influence on the development of modern botany there. The Chinese word “cell” became a botanical and anatomical term after it was introduced into Japan, and later inspired the word “细菌” (bacterium) (Shen, 2010). In the wave of the introduction of Western learning from Japan in the early 20th century, Chinese words such as “cell” were passed back from Japan, which in turn had a profound influence on China.

Throughout the book *Chih-wu hsüeh*, Chinese translations of “细胞” other than “cell” included “locule”, “ovary” and so on. Especially, although “cell” was not necessarily translated into as “细胞”, it can be seen that the translator clearly understood the concept of “细胞”, because he could distinguish the different kinds of concepts. Thus, the conclusion (Huang, 2016) that the translator was pessimistic or hesitant about the translation “cell” based on the lowest frequency of Chinese word “细胞” among the Chinese translations of “cell” was untenable.

It has been more than 160 years since the book *Chih-wu hsüeh* was published, and many Chinese terms translated from English have changed to different extents in the course of development. Even for the Chinese terms that are still in use today, the meaning and scope of the denoted concepts may have changed significantly. The Chinese word “细胞” is no exception. Now, it refers especially to the basic unit of the structure and function of organisms, and has almost completely lost the other meanings given in *Chih-wu hsüeh*.

Shanlan Li, a well-known mathematician of the late Qing dynasty, participated in the translation and publication of many important scientific textbooks, covering mathematics, mechanics, astronomy, botany and other subjects. He was also the first person to introduce algebra, analytic geometry, calculus, botany and other modern Western sciences into China, promoting the spread of modern science in China. As a well-deserved pioneer of modern Chinese science, Li is worth remembering by people forever.
